# Human Equilibrative Nucleoside Transporter-1 Knockdown Tunes Cellular Mechanics through Epithelial-Mesenchymal Transition in Pancreatic Cancer Cells

**DOI:** 10.1371/journal.pone.0107973

**Published:** 2014-10-14

**Authors:** Yeonju Lee, Eugene J. Koay, Weijia Zhang, Lidong Qin, Dickson K. Kirui, Fazle Hussain, Haifa Shen, Mauro Ferrari

**Affiliations:** 1 Department of Nanomedicine, Houston Methodist Research Institute, Houston, Texas, United States of America; 2 Department of Radiation Oncology, M. D. Anderson Cancer Center, Houston, Texas, United States of America; 3 Department of Mechanical Engineering, Texas Tech University, Lubbock, Texas, United States of America; 4 Department of Cell and Developmental Biology, Weill Cornell Medical College, New York, New York, United States of America; 5 Department of Medicine, Weill Cornell Medical College, New York, New York, United States of America; The University of Hong Kong, China

## Abstract

We report cell mechanical changes in response to alteration of expression of the human equilibrative nucleoside transporter-1 (hENT1), a most abundant and widely distributed plasma membrane nucleoside transporter in human cells and/or tissues. Modulation of hENT1 expression level altered the stiffness of pancreatic cancer Capan-1 and Panc 03.27 cells, which was analyzed by atomic force microscopy (AFM) and correlated to microfluidic platform. The hENT1 knockdown induced reduction of cellular stiffness in both of cells up to 70%. In addition, cellular phenotypic changes such as cell morphology, migration, and expression level of epithelial-mesenchymal transition (EMT) markers were observed after hENT1 knockdown. Cells with suppressed hENT1 became elongated, migrated faster, and had reduced E-cadherin and elevated N-cadherin compared to parental cells which are consistent with epithelial-mesenchymal transition (EMT). Those cellular phenotypic changes closely correlated with changes in cellular stiffness. This study suggests that hENT1 expression level affects cellular phenotype and cell elastic behavior can be a physical biomarker for quantify hENT1 expression and detect phenotypic shift. Furthermore, cell mechanics can be a critical tool in detecting disease progression and response to therapy.

## Introduction

Pancreatic adenocarcinoma (PDAC) is one of the most lethal human cancers with an extremely poor prognosis [1]. PDAC has low survival rate, even after complete resection of the tumor, which is the only chance for cure. Unfortunately, most of tumors are unresectable and metastatic. Thus, chemotherapy and/or radiotherapy are the only options [2], [3]. Gemcitabine (2′,2′-difluorodeoxycytidine) is one of efficient anticancer agents for pancreatic cancer [1]. It is a cytotoxic pyrimidine deoxynucleoside analogue that is transported into the cellular compartment through the primary transport protein, human equilibrative nucleoside transporter-1 (hENT1), and eventually inhibits DNA replication. The hENT1 expression level in pancreatic cancer cells has previously been correlated to therapeutic efficacy where cells with higher hENT1 expression were shown to respond better to gemcitabine. Furthermore, cellular level studies have also shown that pancreatic cancer cells with low hENT1 expression are highly resistant to gemcitabine [4]. Moreover, clinical studies have established that hENT1 expression affect how patients respond to treatment where patients whose tumor expressed low hENT1 biomarker responded poorly to gemcitabine therapy [3], [5].

Pancreatic cancer cells that acquire gemcitabine-resistance are characterized by epithelial-mesenchymal transition (EMT) phenotype and show distinct morphological changes from epithelial to spindle-shaped and increasing cellular motility [6], [7]. EMT is a biological process that polarized epithelial cells shift to a mesenchymal-like phenotype through multiple biochemical changes. This phenotypic transition is characterized by loss of cell-cell adhesion and dynamic changes in the structure of the cytoskeleton which cause cells to detach from epithelium and to gain the ability to migrate to distant sites [8], [9]. Thus, in this study, we hypothesized that modulation of hENT1 expression levels in pancreatic cancer cells may alter their physiological characteristics as it may induce phenotypic shift by inhibiting gemcitabine uptake. In addition to biochemical methods to identify cellular physiological changes, understanding cell mechanics can provide new biological insights. Recent studies reveal that mechanical properties of cells provide crucial information to understand various biophysical behaviors that include cell shape, motility, and cell adhesion that generate a cascade of biochemical signals that are critical for biological responses [10]. The mechanical signatures of cells can be an important tool in various aspects: (1) Identification of cancer cells from normal cells based on their relatively lower stiffness [11]; (2) Anticipation of a metastatic potential of cancer cells as cellular stiffness inversely correlates with migration and invasion [12]–[14]; (3) Recognition of phenotypic shifts associated with alteration in intracellular structure and motility in cancer cells by measurement of increases or decreases in elastic modulus [11], [15]–[18]. Although there is no direct evidence that hENT1 is related to phenotypic events in pancreatic cancer cells, we can speculate that hENT1 expression may modulate cellular biophysical behaviors based on the close correlation between hENT1 expression and gemcitabine sensitivity. The cellular phenotypic shift from gemcitabine resistant cells established by culturing cells in serially increasing concentration of gemcitabine also supports our hypothesis.

**Figure 1:**
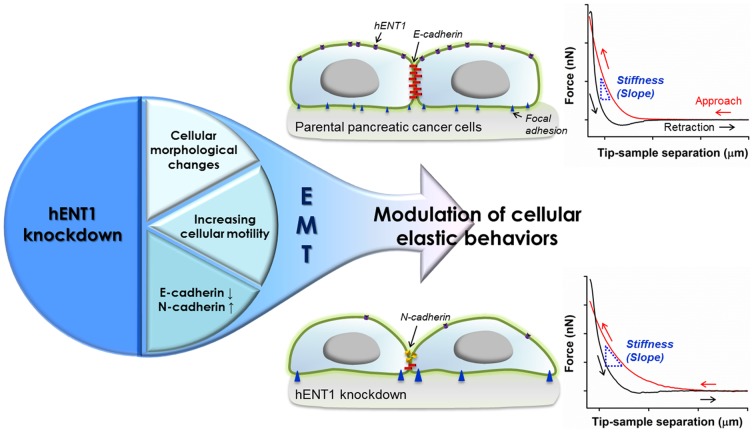
Schematic illustrations describing overall flow of this study. The hENT1 knockdown induces changes in cellular mechanics via EMT accompanied by alterations in E-cadherin and N-cadherin expression levels, cellular morphology, and motility of pancreatic cancer cells. Further, hENT1 knockdown induces decrease in cell stiffness as demonstrated on representative force separation curves obtained from Panc 03.27 cells (upper graph from a parent cell; the second graph from a hENT1 knockdown cell) using AFM.

In this study, two different methods, AFM and a microfluidic platform, were used to evaluate how modulation of hENT1 expression level influences on stiffness of pancreatic cancer cells. Then the accompanying morphological alterations, cytoskeleton rearrangements, cellular motility, and changes in expression levels of EMT markers to investigate cellular phenotypic shift were characterized (Figure 1). Together our results on hENT1 expression level and cell stiffness correlate very well with the mechanistic alterations of intracellular cytoskeletal structure, cellular motility, and suggest that cellular elastic properties can estimate hENT1 expression level as well as phenotypic shift.

## Materials and Methods

### Cell culture

All cell lines were purchased from American Type Culture Collection (ATCC, Manassas, VA). The AsPC-1, BxPC-3, MIA Paca 2, and Panc-1 cells were grown in Dulbeccos Modified Eagles Medium (DMEM, Thermo Scientific Hyclone, Waltham, MA) with 10% fetal bovine serum (FBS, ATLAS Biologicals, Fort Collins, CO), Capan-1 in DMEM with 20% FBS, and Panc 03.27 in RPMI 1640 (Thermo Scientific Hyclone, Waltham, MA) with 15% FBS and human recombinant insulin (10 units/ml, Sigma Aldrich, St. Louis, MO) in the presence of 5% CO_2_ at 37°C.

### hENT1 knockdown with siRNA transfection

The cells were cultured in 6 cm cell culture dish at a density of 5×10^5^ cells/dish. After washing with PBS, cells were treated with INTERFERin (Polyplus transfection™) containing siRNA against hENT1 (SMARTpool: ON-TARGET plus SLC29A, Dharmacon, Inc., Pittsburgh, PA) or negative siRNA (Silencer Negative Control No. 1 siRNA, AM4635, Invitrogen, Grand Island, NY) at a concentration of 50 nM in Opti-MEM (Invitrogen, Grand Island, NY). After 24 hours transfection, cells were washed twice with PBS and incubated for 3 days.

### Western blotting analysis

Cells were lysed with RIPA Pierce buffer (Thermo Scientific, Waltham, MA) with protease inhibitor cocktail (Thermo Scientific, Waltham, MA). Cell lysates (10 µg) with loading buffer (LDS sample buffer Non-reducing, Thermo Scientific, Waltham, MA) were heated for 5 min at 95°C. Cell lysates and protein ladder (Xpert 2 Prestained Protein Marker, GenDEPOT) loaded on polyacrylamide gels (Any kD Mini- PROTEAN TGX Precast Gel, Bio-Rad, Hercules, CA) and transferred to Nitrocellulose Membrane (Bio-rad, Hercules, CA). The blots were blocked with blocking buffer, TBST (20 mM TRIS pH 7.6/150 mM NaCl/0.05% Tween 20) containing 5% milk for an hour. The blots were incubated overnight with TBST and 5% milk containing primary antibodies at certain ratios: anti-hENT1 antibody (1∶1000, Abcam, Cambridge, MA); anti-E-cadherin antibody (1∶1000, Abcam, Cambridge, MA); anti-N-cadherin antibody (1∶500, Abcam, Cambridge, MA); cytokeratin 18 (1∶1000, Cell signaling technology, Danvers, MA); Lamin A/C (1∶1000, Cell signaling technology, Danvers, MA); anti-GAPDH antibody (1∶2000, Cell signaling technology, Danvers, MA). After washing three times with TBST, the blots were incubated with TBST and 5% milk containing secondary antibodies, anti-mouse IgG, HRP-linked antibody (1∶5000, Cell signaling technology, Danvers, MA) or anti-rabbit IgG, HRP-linked antibody (1∶5000, Cell signaling technology, Danvers, MA), for an hour at room temperature. Then, the blots placed onto a mixture of Pierce ECL western blotting substrate (Thermo Scientific, Waltham, MA) and Lumigen TMA-6 (Lumigen, Inc., Southfield, MI), and proteins were detected.

### Immunofluorescence

Pancreatic cancer cells were seeded in 6-well plates containing sterilized, collagen I (Invitrogen, Grand Island, NY)-coated coverslips (22×22 mm, Corning) at a density of 2×10^5^ cells/well. The cells were prepared with three groups: 1) control (without treatment); 2) scramble (transfected with negative control siRNA); and 3) hENT1 knockdown. The cells were fixed with 4% paraformaldehyde (Affymetrix, Santa Clara, CA) for 30 min at room temperature and then permeabilized with 2% Triton X-100 (Sigma Aldrich, St. Louis, MO) for 10 min. Following blocking with 2% bovine serum albumin (Calbiochem) for 1 hour, the cells were incubated overnight with anti-Vinculin antibody (1∶200, Abcam, Cambridge, MA), anti-E-cadherin (1∶200), anti-N-cadhrein (1∶100), anti-cytoketarin 18 (1∶200), or anti-Lamin A/C (1∶200) at 4°C with gentle shaking. Then cells were washed twice with PBS for 10 min and incubated with Alexa 488 or 594-conjugated secondary antibodies for an hour at room temperature. After washing with PBS, for F-actin staining, cells were incubated with Alexa Fluor 488 Phalloidin (1∶250, Invitrogen, Grand Island, NY) for 30 min at room temperature. Then, the nuclei were stained using Hoechst 33342 (Molecular probes, Life Technologies, Grand Island, NY) for 10 min. The cells were monitored by confocal laser scanning microscope (CLSM, Olympus FluoView FV1000).

### AFM measurements

Cellular stiffness was measured by the force-curve technique on a Bioscope (Bruker Corporation, Billerica, MA). The cells were cultured on 6 cm cell culture dish and all measurements were performed in culture medium at 37°C. The AFM was equipped with an inverted light microscope (Olympus IX81) so that the cells were constantly monitored. To minimize cell damage, silica microparticle (diameter: 5 µm) modified silicon nitride cantilevers (Novascan Technologies, Inc., Ames, IA) with approximate spring constant values of ∼0.06 N/m were employed in all AFM experimentations. The exact spring constant value was measured by the thermal tuning method. Probes were positioned at the cells' nuclei proximities under optical control, and force curves were acquired at a sampling rate of 1 Hz. The Young's modulus, E, was calculated from obtained force curves based on the Hertz model (Eq. 1) using Nanoscope analysis program from Bruker corporation.
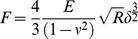
(1)where F = force, E = Young's modulus, ν = Poisson's ratio (ν = 0.5, in this study), R = radius of the indenter (R = 2500 nm, in this study), and δ = indentation depth.

To obtain Young's modulus, two independent experiments were performed and force curves from at least 50 cells were collected in each experiment.

### MS-chip design and fabrication

Silicon wafers (4 inch) from Corning Inc. SU-8 2015 photoresist, and SU-8 developer from MicroChem Corp. and polydimethylsiloxane (PDMS RTV615) from Momentive Performance Materials Inc. were used. The microchip pattern was designed with AutoCAD software (Autodesk Inc.) and then printed out as 10-µm resolution chrome masks by Photo Science Inc. The photomask pattern was first translated into a microstructure on a 4-in silicon wafer using SU-8 2015 photoresist, which is a mold for casting PDMS materials. Briefly, the mold was prepared by spin coating SU-8 2015 photoresist onto a silicon wafer and crosslinking by UV for 180 seconds. Subsequently, the designed pattern was developed using SU-8 developer (Microchem Corp.) and cleaned with isopropyl alcohol and nitrogen gas. The holes for the inlets and outlets were punched using needles. The PDMS layer was cleaned by rinsing with isopropyl alcohol and deionized water, and dried with nitrogen gas. After treatment with oxygen plasma, the PDMS layer was bonded immediately to a 75×50-mm glass slide. Finally, the bonded device was baked for 2 h at 80°C.

### On-chip cell separation

The channels in MS-chip and Tygon tube connected to the chip inlet were wetted with PBS and then kept with 0.5% BSA in PBS for 1 hour. BSA blocks the surface and further prevents nonspecific adhesion of cells to PDMS. Membranes of control and hENT1 knockdown cells were stained using Alexa Fluor 594 or 488-conjugated wheat germ agglutinin (Invitrogen, Grand Island, NY), respectively. The cell mixture in equal amounts at a final density of 1×10^5^ cells/ml was prepared. Suspended cells were then applied to the MS-chip via a Tygon tube. During the experiment, compressed nitrogen gas was applied to the cell suspension at a pressure of ∼10 psi (69×10^3^ Pa). A typical separation lasted ∼15 min, and average flow rate was controlled at 1–2 mL/h. Cells applied to the MS-chip were imaged by fluorescence microscopy (IX81, Olympus). Number of cells retained on chip after separation was counted by Image J.

### *In vitro* scratch assay

The scratch assay was performed on either native cells (control) or transfected cells (scramble and siRNA against hENT1) to study the effect of hENT1 knockdown on cell migration. Capan-1 or Panc 03.27 cells were seeded into 24-well plate. When the cells are approximately 50–60% confluent, cells were washed with PBS and then transfected with INTERFERin containing siRNA against hENT1 or negative siRNA at a concentration of 50 nM in Opti-MEM. After 6 hours transfection, cells were washed twice with PBS and incubated for 2 days. A scratch of the cell monolayer was created by using a pipet tip. Then, the plates was washed and replaced with the desired medium. The time-lapse microscope (EVOS FL Auto Imaging System, Life Technologies) with chamber (95% Air and 5% CO_2_) and temperature control (37°C) was used for acquiring the images from the same field automatically for 18 hours. The images acquired were analyzed quantitatively by using Image J.

### Statistical analysis

The data are expressed as mean ± standard deviation. Statistical significance was identified by two-way ANOVA analysis using GraphPad Prism 5.0. A P value<0.05 was considered to be statistically significant.

## Results and Discussion

**Figure 2:**
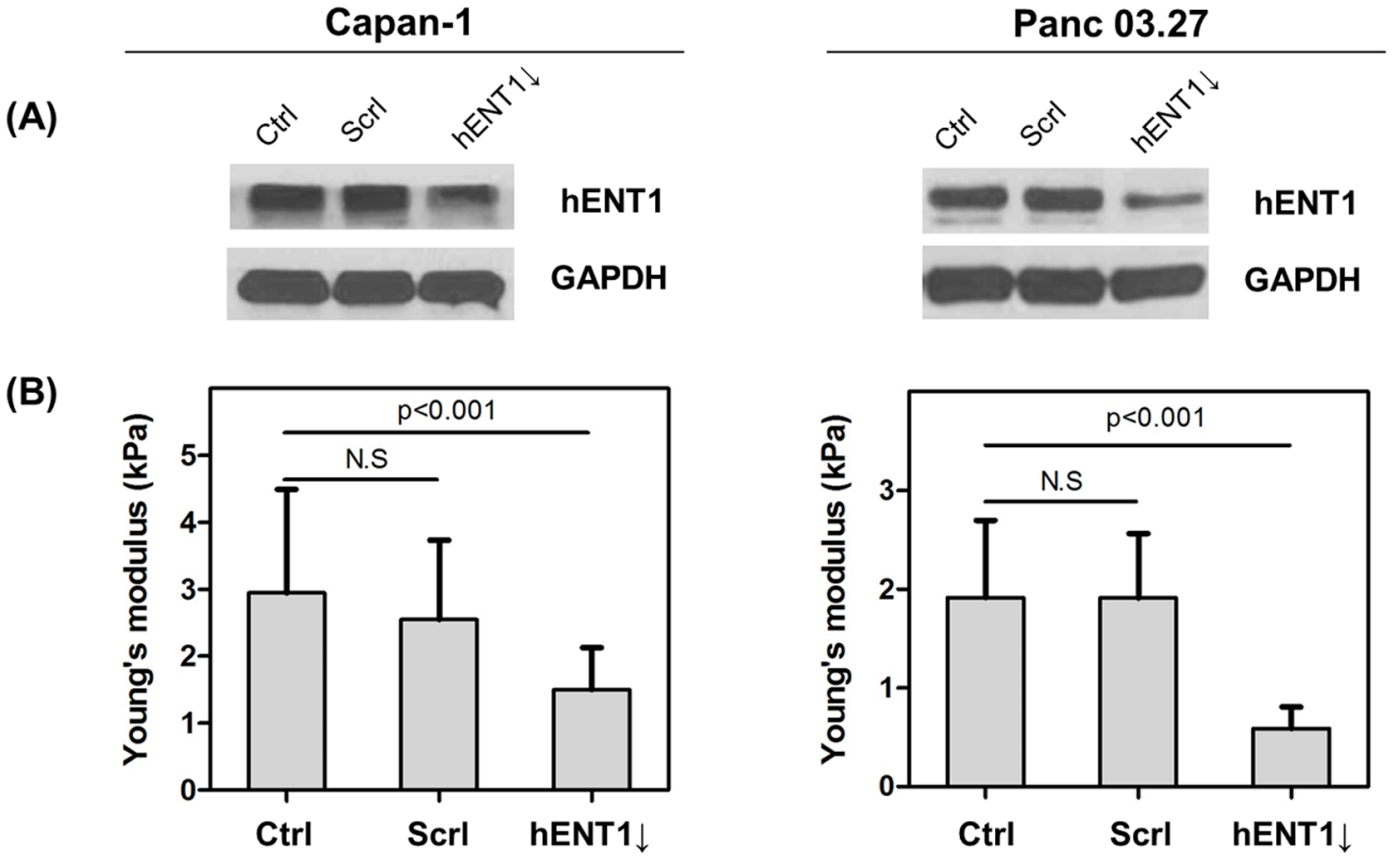
Establish hENT1 knockdown pancreatic cancer cell lines. (A) Western blots of hENT1 (55 kDa) and GAPDH (37 kDa) in Capan-1 (left) and Panc 03.27 cells (right) without treatment (ctrl) or after treatment of negative siRNA (scrl) or hENT1 siRNA (hENT1↓), (B) Bar histograms show Young's modulus of control, negative siRNA transfected, and hENT1 knockdown cells. (N.S: statistically not significant).

To investigate the correlation between hENT1 expression level and cell mechanics, two types of pancreatic cancer cell lines, Capan-1 and Panc 03.27 with relatively higher hENT1 expression (Figure S1 in File S1), were chosen. By siRNA transfection, hENT1 downregulated cells were established (Figure 2). We then measured changes in cell mechanics by two different methods, AFM and microfluidic separation. AFM measures elastic properties of living cells through a direct mechanical interaction between a probe and cell surface. Cell stiffness affects the extent of cantilever deflection upon interaction with the surface of adherent cells [19]. A live cell indentation induces deformation of cell compartments that include membrane, cytoskeletons, nucleus, and various organelles. Thus, the elastic properties of cells result from the aggregated effects of deforming numerous cellular components. We used a silica microparticle (diameter: 5 µm) modified cantilever (Figure S2 in File S1) to reduce cell membrane damage during contact and skew the dataset differently than a sharp tip. To optimize indentation force, we applied force up to 10 nN as shown in Figure S2 in File S1. A constant Young's modulus of Panc 03.27 cells was obtained within the indentation forces up to 200 pN, indicating the measurement of cellular stiffness using AFM is only valid at small deformations of the living cells. Both cell lines showed a similar trend under the same indentation force, i.e. 100 pN. The control cells and cells transfected by negative siRNA were significantly stiffer than hENT1 knockdown cells (Figure 2 and Figure S3 in File S1). The average cell stiffness of both control cells is 2.95±1.55 kPa (Capan-1) and 1.91±0.78 kPa (Panc 03.27); however, that of hENT1 knockdown cells showed significantly decreased Young's moduli with values of 1.50±0.63 kPa (Capan-1) and 0.59±0.22 kPa (Panc 03.27).

**Figure 3:**
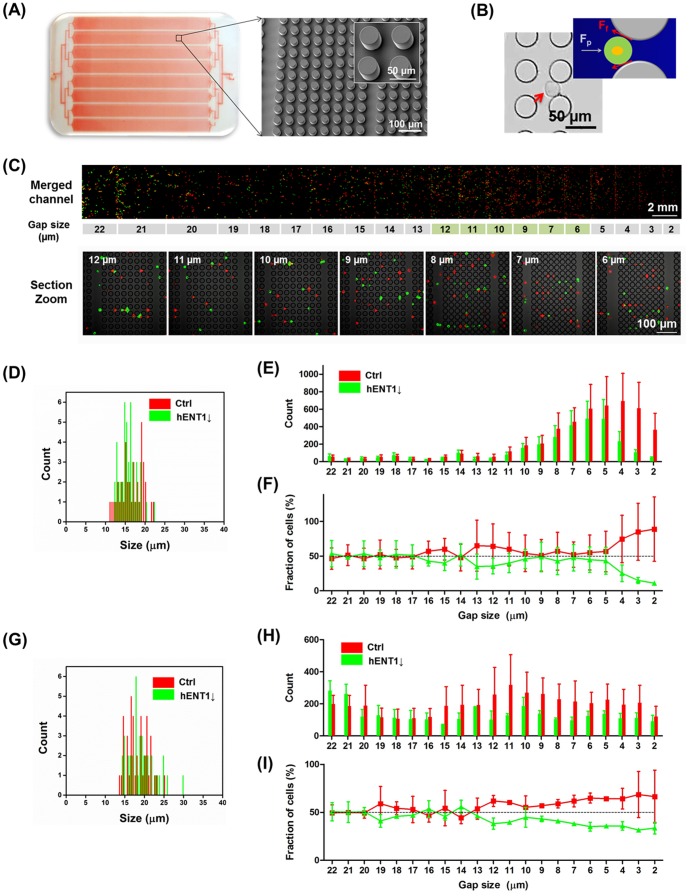
Compare deformability of control and hENT1 knockdown cells using a microfluidic separation chip. (A) Photograph (left) of microfluidic separation chip (MS-chip) visualized by red dye inside and a representative scanning electron microscopic (right) image of the post array (diameter of pillar: 35 µm, height of pillar: 30 µm). (B) Bright field image and scheme of cells flowing in MS-chip when the cell diameter is smaller than pillar gap size (red arrow indicates cells). It is assumed here that friction is due only to shear stress (F_p_: pressure force against the applied pressure (10 psi in this study), F_f_: friction force). (C) The fluorescence image of one of channels among eight shows retained Panc 03.27 cells in the MS-chip after separation of Panc 03.27-ctrl (red fluorescence) and Panc 03.27-hENT1 knockdown (green fluorescence). Representative higher magnification confocal microscopic images of the MS chip (gap size from 12 µm to 6 µm) show the efficiency of separation through gaps. Size distribution of cells (D: Capan-1, G: Panc 03.27 cells). Statistical analysis of cells (E: Capan-1, H: Panc 03.27) and fraction ratio of cells (F: Capan-1, I: Panc 03.27) retained on chip. Values represent mean ± standard deviation.

Since AFM is limited to local measurement of cell mechanics, we also evaluated cellular deformability using microfluidic separation method [20] to corroborate AFM findings. A mechanical separation chip (MS-chip, Figure 3A) was designed with artificial microbarriers in combination with hydrodynamic force to separate deformable from stiff cells [21]. To demonstrate the capacity of the MS-chip to separate cells based on deformability, we tested the separation of a mixture containing two different cells: control and hENT1 knockdown. Membrane of control and hENT1 knockdown cells was stained by using Alexa Fluor 594 and 488 conjugated wheat germ agglutinin (WGA), respectively. As shown in Figure S4 in File S1, the effect of WGA on cell stiffness is negligible. The proportion of the two cell lines varied along the length of the chip. As shown in Figure 3D and 3G, diameters of both cells, control and hENT1 knockdown, were similar. The cell mixture in equal amounts at a density of 1×10^5^ cells/mL was injected to MS-chip and flowed for 15 min. The gaps between posts array in this designed MS-chip range from 22 µm to 2 µm. The channels avoid obstruction that occurs in repeating arrays of posts which regulate and equalize hydrodynamic pressure throughout the chip. It is assumed that there is no physical contact between PDMS pillars and cells because the PDMS is coated with BSA. The shear stress is a main factor to interact with cells. When the gap size of post arrays is bigger than cell diameter, pressure force (F_p_, 10 psi in this study) is the only force acting on cells. If cell diameter is the same or larger than the gap size, friction force (F_f_) opposes pressure force (Figure 3B). If cells are stiffer, they push harder on the post arrays, and then F_f_ is significantly increased. Figure 3C shows Panc 03.27 cells trapped on MS-chip imaged by fluorescence microscopy; red fluorescence shows control cells and green fluorescence shows hENT1 knockdown cells. At the inlet, equal number of red control Panc 03.27 (or Capan-1) cells and green hENT1 knockdown Panc 03.27 (or Capan-1) cells were observed (Figure 3C and 3E–I). In this region, channels were much wider that the diameter of cells, resulting in no significant separation of flexible and stiff cells. Due to smaller diameter of Capan-1 cells (average diameter: 15 µm), the separation was achieved with 8 µm gap size of post arrays. But, in case of Panc 03.27 cells (average diameter: 19 µm), the major separation occurs at 12 µm. In these cell lines, cells in the control group were consistently trapped while hENT1 knockdown cells passed through the gaps more frequently. These experiments demonstrate the overall efficiency of separation of the two different phenotypes with similar diameter in the MS-chip. Thus, we conclude both control cells are relatively stiffer than hENT1 knockdown cells, and these results are in agreement with AFM findings.

**Figure 4:**
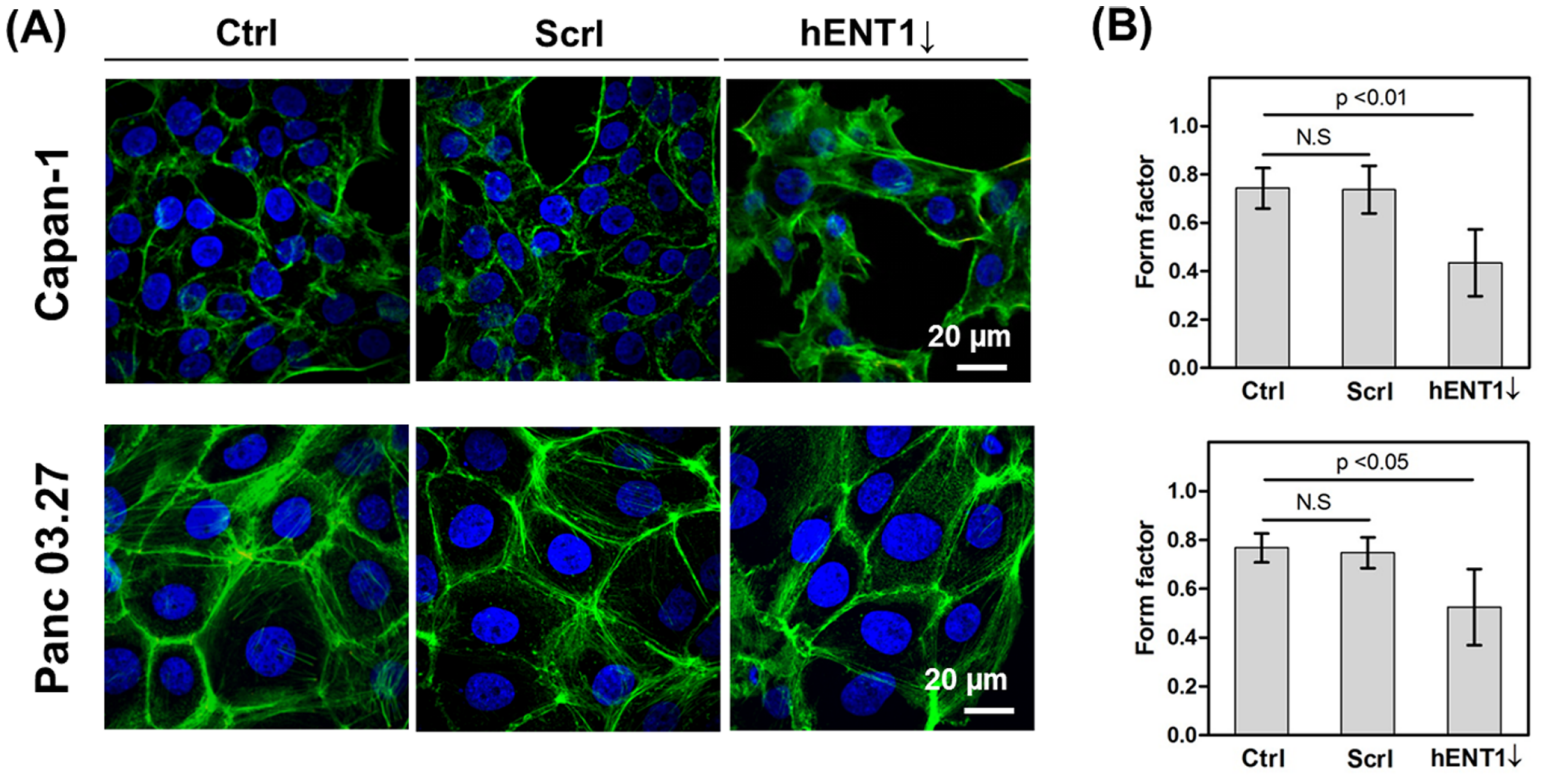
Quantify cell roundness. (A) Representative confocal micrographs of pancreatic cells (Top panel: Capan-1, Bottom panel: Panc 03.27) showing F-actin distribution, (B) form factor (f = 4πa/p^2^; a: area; p: perimeter) analyzed by Image J (Capan-1 cells, ctrl: n = 37, scrl: n = 59, hENT1↓: n = 29; Panc 03.27, ctrl: n = 36, scrl: n = 28, hENT1↓:n = 28, N.S: statistically not significant).

To understand how hENT1 influences cell mechanics, physiological changes in cells after hENT1 knockdown were studied by confocal microscopy. We observed that cell morphological changes associated with hENT1 knockdown. As shown in Figure 4, cells were visualized using confocal laser scanning microscope and the cell roundness was quantitatively analyzed by calculating form factors (FF). The FF is obtained by the equation, 4π(area)/(perimeter)^2^, which gives a value of 1 for a perfectly circular perimeter and decreasingly smaller positive values for less circular perimeters [22]. Capan-1 and Panc 03.27 cells are epithelial type and they are closely attached to each other via intercellular adhesion complexes. They have squarish shapes with FF values of 0.74±0.08 and 0.77±0.06, respectively (Figure 4B). In contrast, both cells after hENT1 knockdown became elongated with much lower FF with values of 0.44±0.14 and 0.52±0.16, respectively.

**Figure 5:**
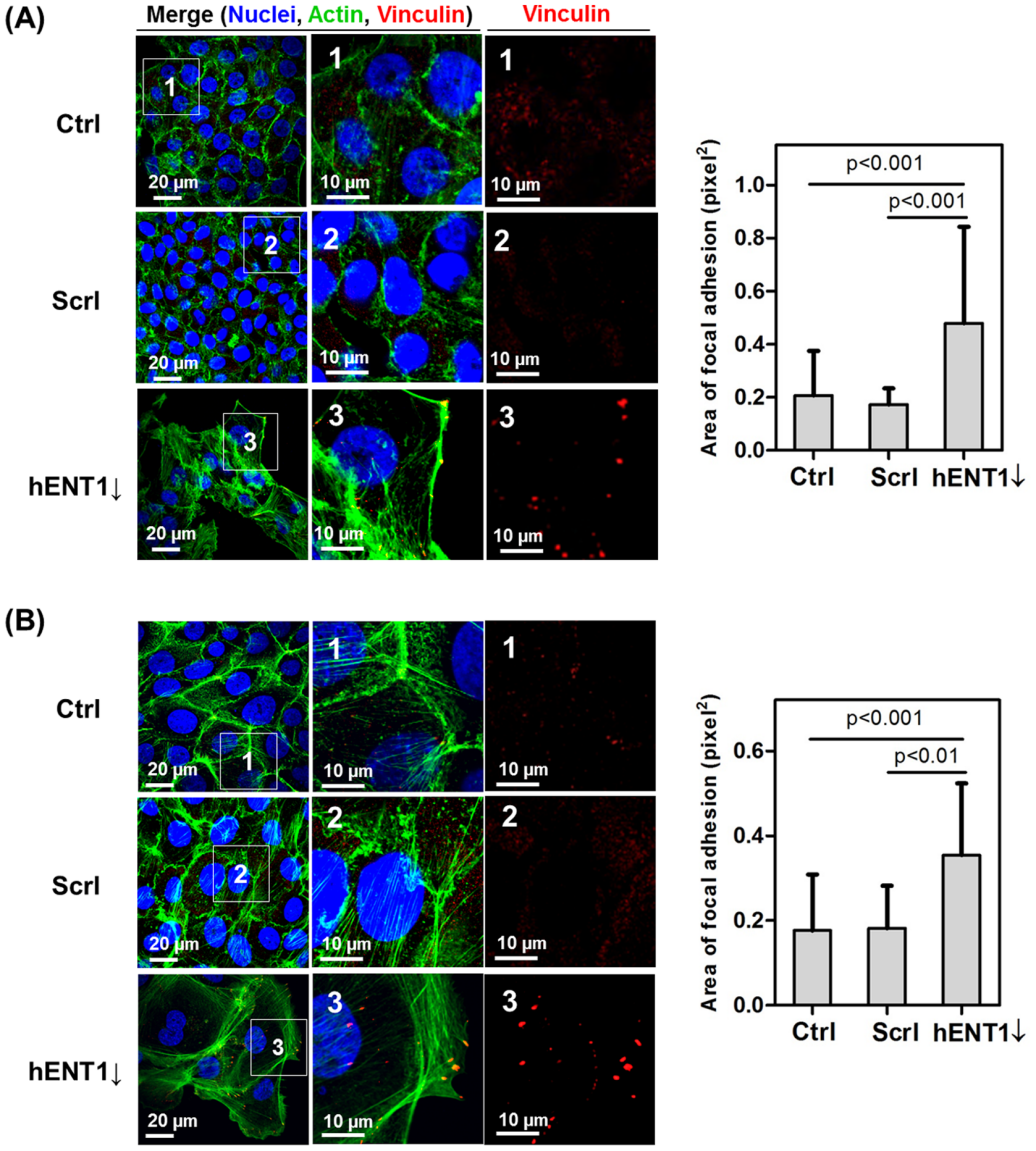
Analysis of focal adhesion area. Confocal micrographs (z-stacks of basal plane, 0–1.5 µm) showing vinculin (red) and F-actin (green) in (A) Capan-1 and (B) Panc 03.27 cells. Area of focal adhesion (pixel^2^) is analyzed by Image J.

Further, the effect of hENT1 knockdown on organization of cytoskeletons was evaluated. We stained for vinculin, which controls the formation of focal adhesions (FAs) by directly interacting with actin and other proteins that are involved in FA formation [23]. FAs, sites of adhesions between the cell and the extracellular matrix (ECM), and stress fibers at the margin have clear roles in moving the cell forward [23], [24]. Studies have reported that FA size is correlated with cell speed; larger FAs found in more elongated and faster-moving cells [25], [26]. In this study, we analyzed redistribution of F-actin and changes in focal adhesion area of hENT1 knockdown cells (Figure 5). Actin fibers in both control groups were mostly concentrated around the cell periphery, and small, nascent FAs were observed. But, after hENT1 knockdown, there was an increase in the number of stress fibers as well as measurable focal adhesion areas. These results suggest increased cellular motility in both cell lines.

**Figure 6:**
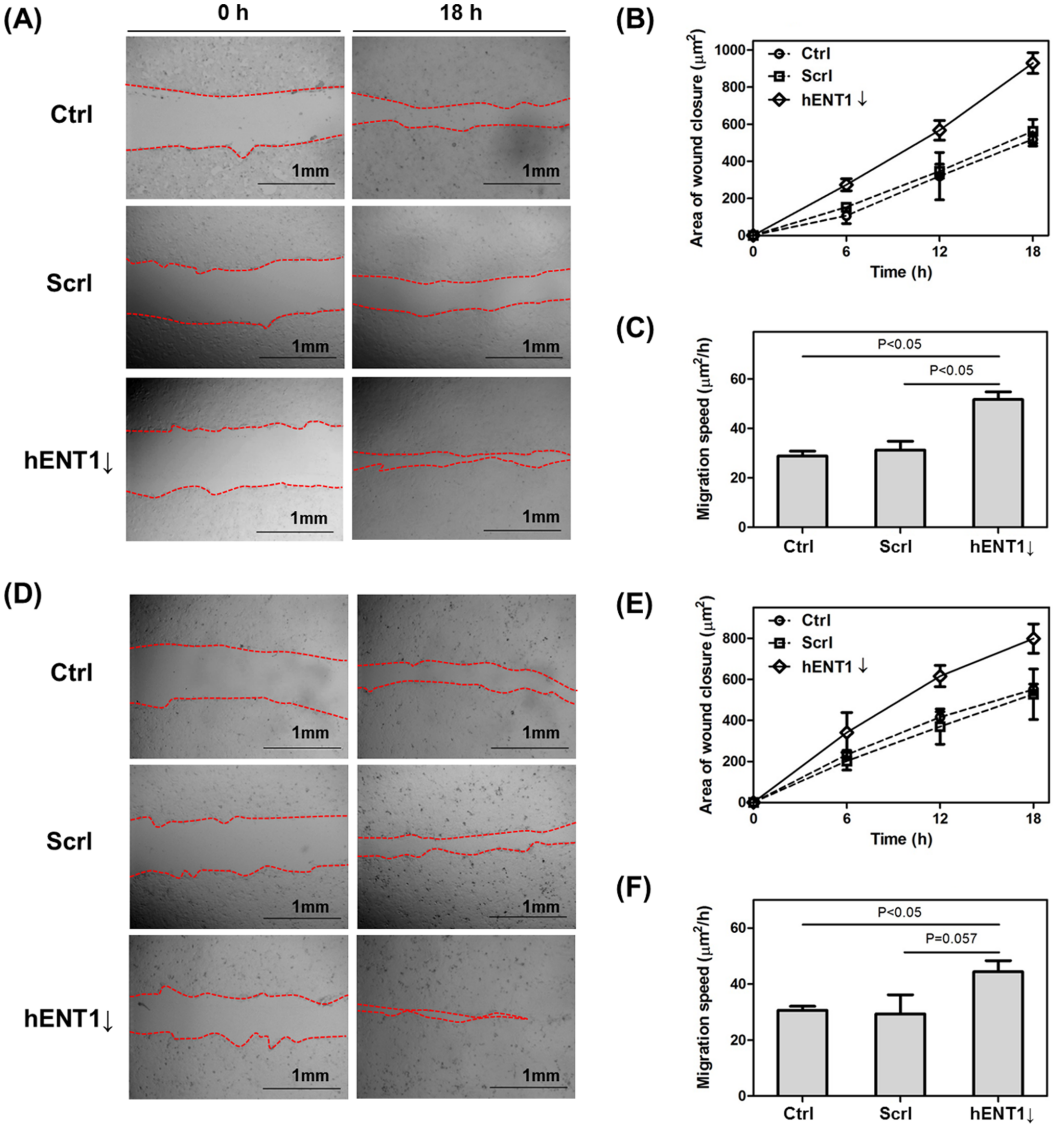
Downregulation of hENT1 promote cell migration. The scratches were introduced to monolayer (A) Capan-1 and (D) Panc 03.27 cells. Photographs were taken immediately after wound induction for 18 hours. The wound sealing areas of (B) Capan-1 and (E) Panc 03.27 cells were calculated using Image J and compared with control, scramble siRNA transfected, and hENT1 knockdown cells. Migration speed of (C) Capan-1 and (F) Panc 03.27 cells was calculated.

In addition, in order to understand effect of hENT1 knockdown on cell motility, we examined the effect of downregulation of hENT1 on cell migration. A wound scratch was introduced in confluent cell monolayer, and then the wound sealing was observed for 18 hours (Figure 6). The area of wound closure was calculated by using Image J and the migration speed (area of wound closure (µm^2^)/hour) was calculated as shown in Figure 6. The wound sealing area and migration speed of both of control cells and cells transfected with scramble siRNA are similar. But hENT1 suppressed Capan-1 or Panc 03.27 cells migrate 1.8 (1.7) or 1.5 (1.5) –fold faster than control (scramble siRNA transfected) cells, respectively. Thus, we confirm that downregulation of hENT1 induces formation of larger focal adhesions and promotes faster cell migration. Based on cell morphological changes and increased cell motility, it is possible that both of cells are undergoing phenotypic changes, which is associated with loosened adhesion between cells and cytoarchitectural rearrangement. Upon EMT, reorganization of cytoskeletons along with increase in FA dynamics is crucial for cells to leave the epithelium and begin migrating through the ECM [22], [27], [28]. For example, a recent study showed increased migration of post-EMT SKOV-3 cells which was transforming growth factor-β (TFG-β) that generated larger FA mechanical forces than that of pre-EMT cells [16]. TGF-β is reported as EMT inducer in different epithelial cell lines including renal proximal tubular and alveolar epithelial cells [16], [29], [30]. Increased interaction of actin cytoskeleton and intracellular molecules with FAs initiated by the association of integrin α5β1 and fibronectin enhanced cellular motility [16].

**Figure 7:**
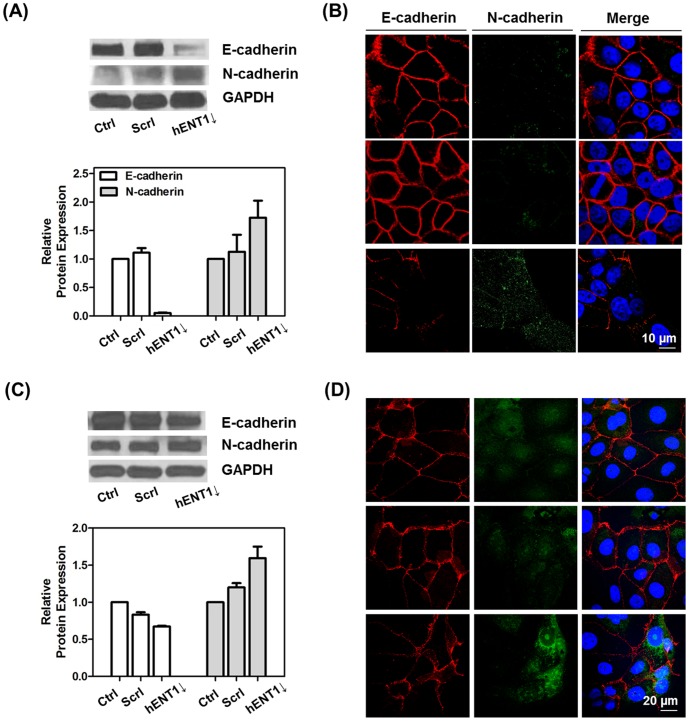
hENT1 knockdown induces changes in EMT markers. Western blots and relative expression levels of E-cadherin (110 kDa), N-cadherin (140 kDa) in (A) Capan-1 and (C) Panc 03.27 cells after treatment with hENT1 siRNA. Columns in the histograms are the mean of two independent experiments (intensity ratio of GAPDH to E-cadherin or N-cadherin). Representative confocal images of E-cadherin (red fluorescence) and N-cadherin (green fluorescence) expressions in (B) Capan-1 and (D) Panc 03.27 cells.

In order to confirm those physiological changes induced by hENT1 knockdown are related to EMT, we analyzed changes in EMT marker proteins, such as E-cadherin and N-cadherin, after hENT1 knockdown. E-cadherin is one of cell-cell adhesion molecules. That maintains cell-cell contacts and epithelial architecture. Alternation of expression of cadherins from E-cadherin to N-cadherin, which is primarily expressed in mesenchymal cells, occurs during EMT. This alternation leads to a drastic change in the adhesive properties of cells, as it loses its affinity for epithelial neighbors and gains affinity for mesenchymal cells, which are nonpolarized, lack intercellular junctions, and have a unique spindle-like shape [27], [31], [32]. After hENT1 downregulation, both cell lines showed low expression of E-cadherin and high expression of N-cadherin, which are a major hallmark of EMT (Figure 7). The loss of E-cadherin and gain of N-cadherin increases cell motility and metastatic potential. Several studies have shown that reduced cell stiffness directly correlates with increased metastatic potential using various in vitro biomechanical analysis methods [11], [17], [33]. Reduction in cellular stiffness modulates that cells are undergoing EMT after hENT1 knockdown as it is demonstrated in Figure 2. However, there are negligible difference in expression levels of intermediate filaments, cytokeratin 18, and nuclear cytoskeleton, lamin A/C in both of cell lines (Figure S5 in File S1).

To confirm our forementioned studies which suggest that EMT can be characterized by changes in cell stiffness, we evaluated phenotypic changes of pancreatic cancer cells after treatment with TGF-β. The TGF-β has been reported in several studies to induce EMT [15], [16], [30]. Therefore, we further examined these results in another experiment using EMT-induced pancreatic cancer cells. Panc 03.27 cells were exposed to TGF-β for 2 days at a concentration of 10 ng/ml to induce EMT, and then measured cellular stiffness. Slightly suppressed E-cadherin and elevated N-cadherin and vimentin expressions indicate that EMT is induced in Panc 03.27 cells (Figure S6A in File S1). Panc 03.27 cells treated with TGF-β were demonstrated by decreased Young's modulus (1.42±0.53 kPa) versus untreated cells (1.91±0.78 kPa). This result suggests that cell stiffness decreases when the cells are undergoing EMT. Recently, there is a report concerning cellular stiffness changes in EMT-induced A549 cells with TGF-β treatment [15]. In their study, they measured local mechanical properties of A549 cells using a sharp DNP cantilever (20 nm tip radius) which has a smaller contact area compared to a microparticle-modified cantilever. Even though there is crucial evidence of cell phenotypic change from epithelial to mesenchymal, i.e., cellular shape changes and increasing stress fibers, are still consistent with our work. The A549 cells treated with TGF-β became stiffer than untreated cells, which is different from our findings. Probably, this conflict result is because they used a sharp tip and the stiffness value of these cells reflects structural rearrangements of F-actin stress fibers in the cells. To demonstrate whether different tip geometry is a critical factor to distinguish cellular compartments, additional experiment with two different AFM cantilevers, a pyramid tip cantilever with 10 nm nominal radius (Figure S7A in File S1) and 5 µm silica bead-modified cantilever (Figure S7D in File S1), were performed. Sequential stiffness maps of Panc 03.27 cells demonstrated that a sharp tip is capable of distinguishing between different cellular compartments (Figure S7B, C in File S1). However, stiffness of overall cells obtained by microparticle-modified cantilever is similar (Figure S7E, F in File S1). The stiffness of cytoplasm region is 2.2-fold higher than nucleus region, when a sharp tip was used; Young's moduli from cytoplasm and nucleus are 68.31±28.00 kPa and 31.10±6.29 kPa, respectively. These results suggest that stiffness obtained from cell membrane and cytoskeleton (cytoplasm region) is higher than that from combination of cell membrane, cytoskeleton nucleus cytoskeleton, and nuclei (nucleus region). A sharp AFM cantilever creates 9.3-fold (nucleus region) and 17-fold (cytoplasm region) increases in the value of the Young's modulus relative to that of the microparticle-modified cantilever under same indentation force at the same loading rate. The Young's moduli from cytoplasm and nucleus areas obtained by using microparticle-modified cantilever are 2.30±0.65 kPa and 1.91±0.82 kPa, respectively. The small contact area of the pyramidal tip is able to identify the mechanical properties of a single cytoskeleton filament, especially in the periphery of a cell. But, the microparticle-modified cantilever has larger contact area compared to a sharp cantilever. Thus, it can indent several cytoskeleton filaments simultaneously and well-organized filaments under cell membrane dissipate the indenting force. These results confirm our hypothesis that different tip geometry influences on detecting specific cellular compartments. The microparticle-modified cantilever is not able to distinguish cellular compartments, however, consistent and reproducible results are obtained from overall cells suggesting this cantilever is suitable to measure cell stiffness compared to a sharp tip.

To further demonstrate that changes in cell mechanical properties upon EMT are universal features of both parental and transformed cells, we evaluated stiffness in six different types of pancreatic cancer cells that classifies as either epithelial-like or mesenchymal-like cells (Figure S8 in File S1). Three cell lines which include AsPC-1, MIA Paca 2 and Panc-1 have vimentin expression and less or no expression of E-cadherin indicating mesenchymal-like cells. As measured by AFM, these cells are relatively softer and have lower hENT1 expression than other three epithelial-like cell lines, BxPC-3, Capan-1, and Panc 03.27. These findings comparing cell stiffness in parental cell lines are consistent to the results where siRNA was used to knockdown hENT1 and induce phenotypic shift. Also, it is consistent with the findings from siRNA transfected cells that mesenchymal-like cells are softer than epithelial-like cells. It is still difficult to fully explain how hENT1 regulates E-cadherin or N-cadherin expression and further cellular stiffness; however, we can conclude that hENT1 expression level is somehow related to cellular stiffness based on results. Also, our results establish a relationship between cell stiffness and EMT whereby cells are undergoing EMT showed reduced stiffness. Those findings are consistent with other studies which have shown that cancer cells from body fluids from patients diagnosed with metastatic tumor are more than 70% softer than the benign cells [11]. Therefore, we suggest that cellular mechanical properties are a critical marker to estimate hENT1 expression and identify phenotypic shift, which is a hallmark in cancer metastasis.

## Conclusions

In this study, we described the role that hENT1 plays in modulating physiological and mechanical properties of pancreatic cancer cells. After downregulation of hENT1, cells became elongated, migrated faster with larger focal adhesion area, and showed altered EMT marker expressions that included downregulation of E-cadherin and upregulation of N-cadherin. These properties are primary signatures associated with phenotypic shift, EMT. In addition, hENT1 knockdown also decreased cellular stiffness which was evaluated with AFM and confirmed by microfluidic platform. These findings suggest that hENT1 knockdown induces EMT in pancreatic cancer cells, results that we corroborated with further measurements in parental pancreatic cancer cells. In conclusion, we have established a novel method to evaluate alterations in cellular biophysical behaviors that result from hENT1 knockdown – a critical drug transporter that has been correlated to patients' response to chemotherapeutic treatment. The ability to predict drug response by evaluating cellular or tumor biophysical properties is a potentially powerful tool that can aid clinicians monitor how patients respond to therapy and may provide predictive capability for personalized treatment.

## Supporting Information

File S1 Figure S1, Western blots of hENT1 (55 kDa) and GAPDH (37 kDa) in pancreatic cancer cells. Figure S2, Young's modulus of Panc 03.27 cells at different indentation force from 10 pN to 10000 pN (inset: Bright-field image showing AFM tip approaching cells and schematic diagram of indentation of microparticle-modified cantilever and cell) (A), Calculated average indentation depth (nm) corresponding to Figure S2A using Eq. (1), and representative force-displacement (f-d) curve from a Panc 03.27 control cell when the indentation force is (C) 100 pN and (D) 1 nN (red: approach curve, black: retract curve) (B). The calculated Young's moduli from (C) and (D) are 1.88 and 5.21 kPa, respectively. Figure S3, Stiffness distribution of (A) Capan-1 and (B) Panc 03.27 cells corresponding to bar histograms shown in Figure 1. The solid line shows Lorentizan distribution. Figure S4, Cellular stiffness of Panc 03.27 cells: Ctrl (without treatment); WGA treated (cell membrane is stained by Alexa Fluor 488 Conjugated wheat germ agglutinin). Young's modulus of cells measured by AFM under same indentation force at 100 pN. Figure S5, Representative confocal micrographs of pancreatic cancer Capan-1 and Panc 03.27 cells showing cytokeratin 18 (green, top panel), Lamin A/C (green, middle and bottom panels), and nuclei (blue) (A). (B) Western blots of Lamin A/C (74, 63 kDa), cytoketarin 18 (46 kDa) and GAPDH (37 kDa) in control, scramble siRNA transfected, and hENT1 knockdown Capan-1 and Panc 03.27 cells. Figure S6, Western blots of E-cadherin (110 kDa), N-cadherin (140 kDa), vimentin (57 kDa), and GAPDH (37 kDa) in untreated and TGF-β treated Panc 03.27 cells (A), (B) Young's modulus of untreated and TGF-β treated Panc 03. 27 cells (concentration of TGF-β: 10 ng/ml, exposure for 2 days). Figure S7, Representative AFM topographic image, deflection image, and corresponding stiffness map of Panc 03.27 cells obtained by using sharp MSCT-C (A, B) and microparticle modified cantilever (D, E). Histograms show (C, F) corresponding stiffness from force volume map (B, E). Figure S8, Young's modulus of cells measured by AFM under same indentation force at 100 pN (A), (B) Western blots of E-cadherin (110 kDa), vimentin (57 kDa), and GAPDH (37 kDa) expressed in six different pancreatic cancer cells.
(DOCX)
