# Supplementary material for: Human Equilibrative Nucleoside Transporter-1 Knockdown Tunes Cellular Mechanics through Epithelial-Mesenchymal Transition in Pancreatic Cancer Cells
Source: PLoS One. 2014 Oct 14;9(10):e107973. doi: 10.1371/journal.pone.0107973 (PMC4196761; doi:10.1371/journal.pone.0107973)
Supplement: File S1 — Figure S1, Western blots of hENT1 (55 kDa) and GAPDH (37 kDa) in pancreatic cancer cells. Figure S2, Young's modulus of Panc 03.27 cells at different indentation force from 10 pN to 10000 pN (inset: Bright-field image showing AFM tip approaching cells and schematic diagram of indentation of microparticle-modified cantilever and cell) (A), Calculated average indentation depth (nm) corresponding to Figure S2A using Eq. (1), and representative force-displacement (f-d) curve from a Panc 03.27 control cell when the indentation force is (C) 100 pN and (D) 1 nN (red: approach curve, black: retract curve) (B). The calculated Young's moduli from (C) and (D) are 1.88 and 5.21 kPa, respectively. Figure S3, Stiffness distribution of (A) Capan-1 and (B) Panc 03.27 cells corresponding to bar histograms shown in Figure 1. The solid line shows Lorentizan distribution. Figure S4, Cellular stiffness of Panc 03.27 cells: Ctrl (without treatment); WGA treated (cell membrane is stained by Alexa Fluor 488 Conjugated wheat germ agglutinin). Young's modulus of cells measured by AFM under same indentation force at 100 pN. Figure S5, Representative confocal micrographs of pancreatic cancer Capan-1 and Panc 03.27 cells showing cytokeratin 18 (green, top panel), Lamin A/C (green, middle and bottom panels), and nuclei (blue) (A). (B) Western blots of Lamin A/C (74, 63 kDa), cytoketarin 18 (46 kDa) and GAPDH (37 kDa) in control, scramble siRNA transfected, and hENT1 knockdown Capan-1 and Panc 03.27 cells. Figure S6, Western blots of E-cadherin (110 kDa), N-cadherin (140 kDa), vimentin (57 kDa), and GAPDH (37 kDa) in untreated and TGF-β treated Panc 03.27 cells (A), (B) Young's modulus of untreated and TGF-β treated Panc 03. 27 cells (concentration of TGF-β: 10 ng/ml, exposure for 2 days). Figure S7, Representative AFM topographic image, deflection image, and corresponding stiffness map of Panc 03.27 cells obtained by using sharp MSCT-C (A, B) and microparticle modified cantilever (D, E). Histogr [file pone.0107973.s001.docx]

**Supporting Information**

**Human Equilibrative Nucleoside Transporter-1 Knockdown Tunes Cellular Mechanics through Epithelial-Mesenchymal Transition in Pancreatic Cancer Cells**

Yeonju Lee,^1^ Eugene J. Koay,^1,2^ Weijia Zhang,^1^ Lidong Qin,^1,4^ Dickson K. Kirui,^1^ Fazle Hussain,^3^ Haifa Shen,^1,4^ and Mauro Ferrari^1,5*^

1. Department of Nanomedicine, Houston Methodist Research Institute, 6670 Bertner Ave., Houston, Texas 77030

2. Department of Radiation Oncology, M. D. Anderson Cancer Center, 1220 Holcombe Blvd., Houston, TX 77030

3. Department of Mechanical Engineering, Texas Tech University, 2703 7^th^ Street, Lubbock, TX 79409

4. Department of Cell and Developmental Biology, 5. Department of Medicine, Weill Cornell Medical College, New York, New York 10065, USA

* Correspondence: mferrari@HoustonMethodist.org

Keywords: Cell stiffness, EMT, hENT1, Pancreatic cancer

**
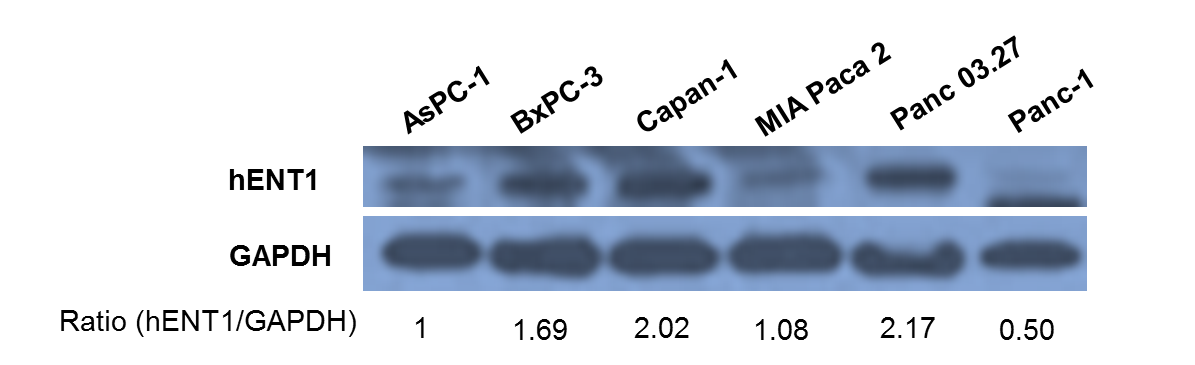
**

Figure S1. Western blots of hENT1 (55kDa) and GAPDH (37 kDa) in pancreatic cancer cells

**Optimization of indentation condition and data processing**


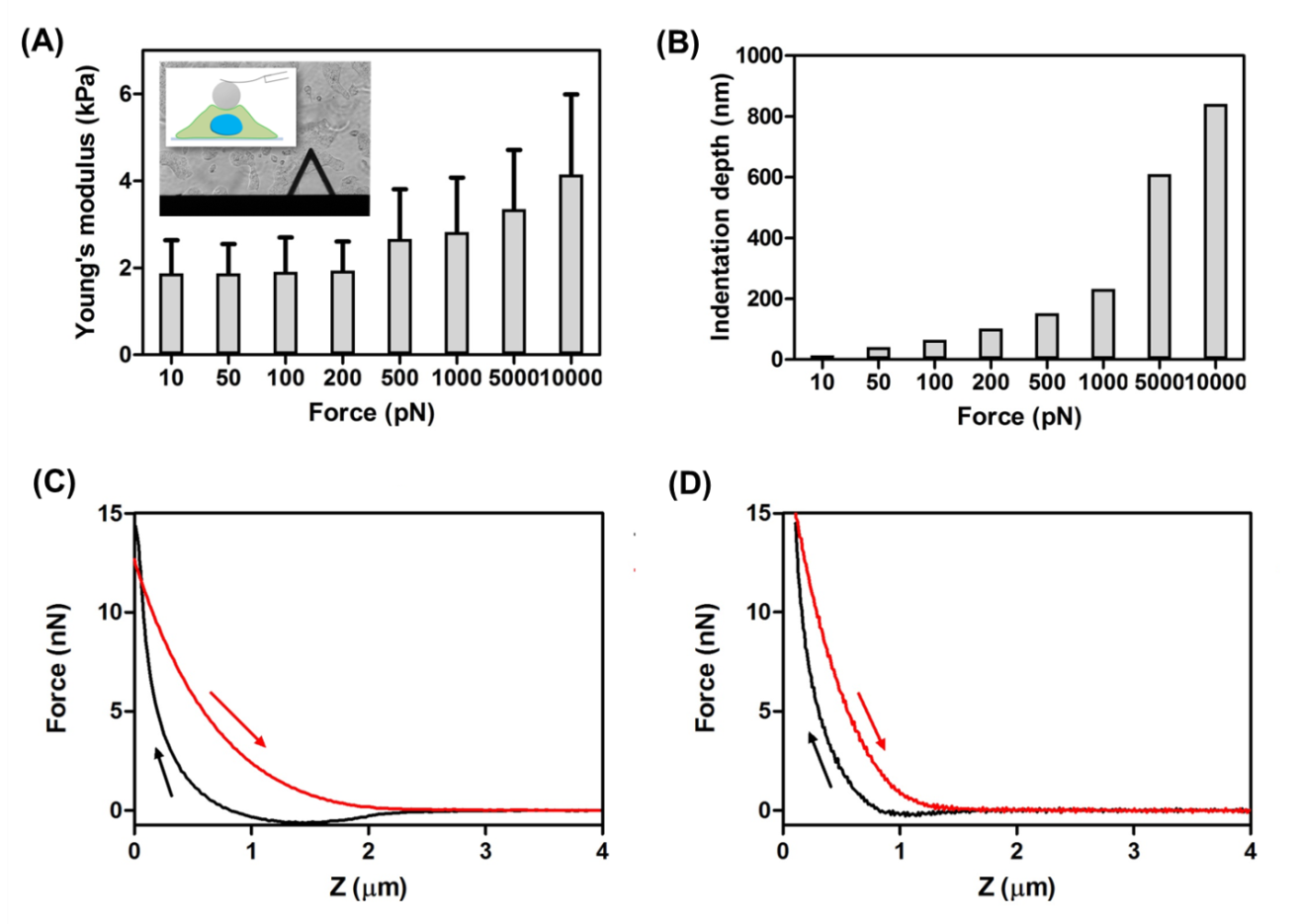
To determine indentation force range which is valid for Hertz model, we applied different indentation forces from 10 pN to 10 nN and obtained Young’s modulus based on Hertz model (Figure S2). Young’s modulus is the intrinsic properties of materials, which is defined by the normal stress divided by linear strain [[1](#_ENREF_1)]. A constant Young’s modulus of Panc 03.27 cells is obtained within the indentation forces up to 200 pN, indicating the measurement of cellular stiffness using AFM is only valid at small deformations of the living cells. According to Eq. 1, indentation depth was calculated as shown in Figure S2C. Additionally, it is expected that reduced nonlinear cellular deformation based on a more homogeneous contact between that cells and the microparticle-modified probe. Moreover, the substrate can utilize effects on the cell that make it appear stiffer, due to height variations of cells, from the cell center to the edge of the cells. Thus, most studies probe cell-stiffness over the central region of the cell an within a certain indentation limit, for example, ~10% of the cell’s height [[2](#_ENREF_2)]. There is an additional concern that the AFM and nanoindentation technique have been developed for relatively hard materials based on Hertz model [[3](#_ENREF_3)]. This model assumes that the materials with a flat surface are elastic, isotropic, homogeneous and linear, following Hooke’s law [[4](#_ENREF_4)].

Figure S2. (A) Young’s modulus of Panc 03.27 cells at different indentation force from 10 pN to 10000 pN (inset: Bright-field image showing AFM tip approaching cells and schematic diagram of indentation of microparticle-modified cantilever and cell), (B) Calculated average indentation depth (nm) corresponding to Figure S2A using Eq. (1), and representative force-displacement (f-d) curve from a Panc 03.27 control cell when the indentation force is (C) 100pN and (D) 1 nN (red: approach curve, black: retract curve). The calculated Young’s moduli from (C) and (D) are 1.88 and 5.21 kPa, respectively.


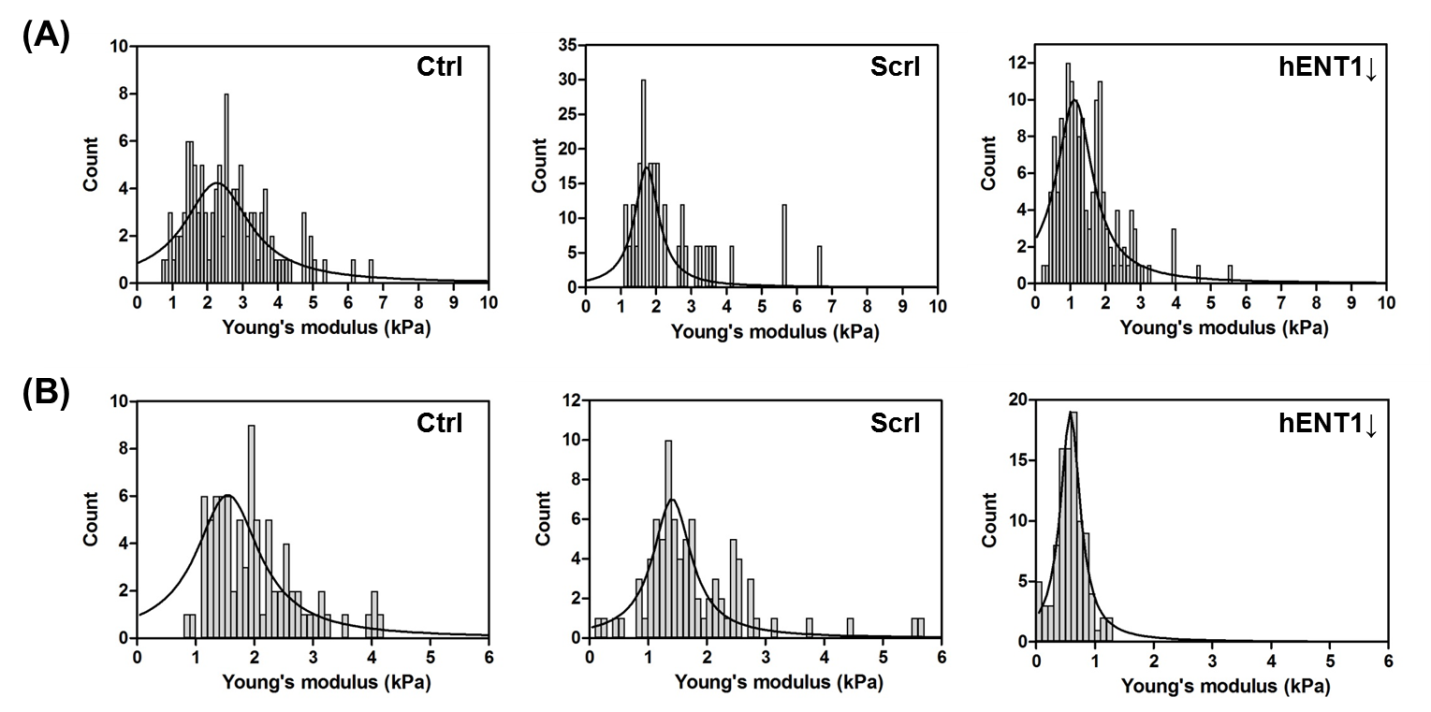


Figure S3. Stiffness distribution of (A) Capan-1 and (B) Panc 03.27 cells corresponding to bar histograms shown in Figure 1. The solid line shows Lorentizan distribution.


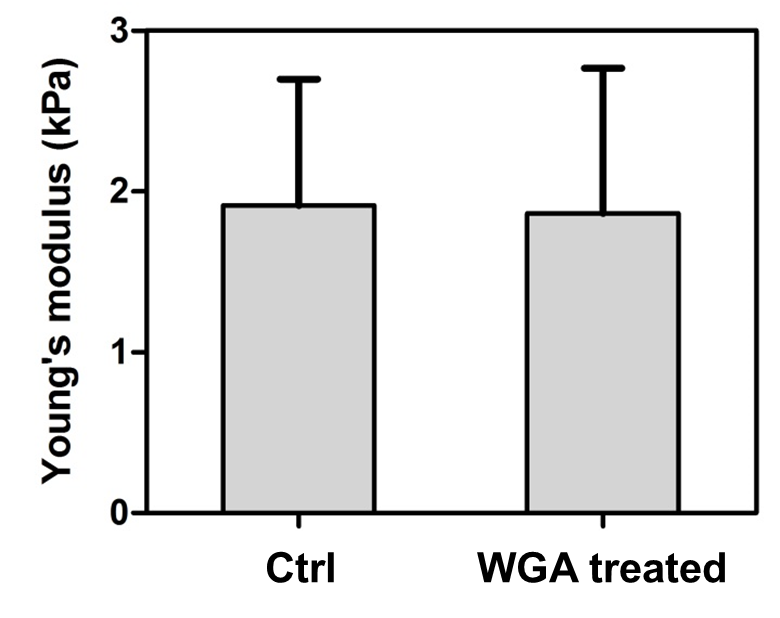


Figure S4. Cellular stiffness of Panc 03.27 cells: Ctrl (without treatment); WGA treated (cell membrane is stained by Alexa Fluor® 488 Conjugated wheat germ agglutinin). Young’s modulus of cells measured by AFM under same indentation force at 100 pN.


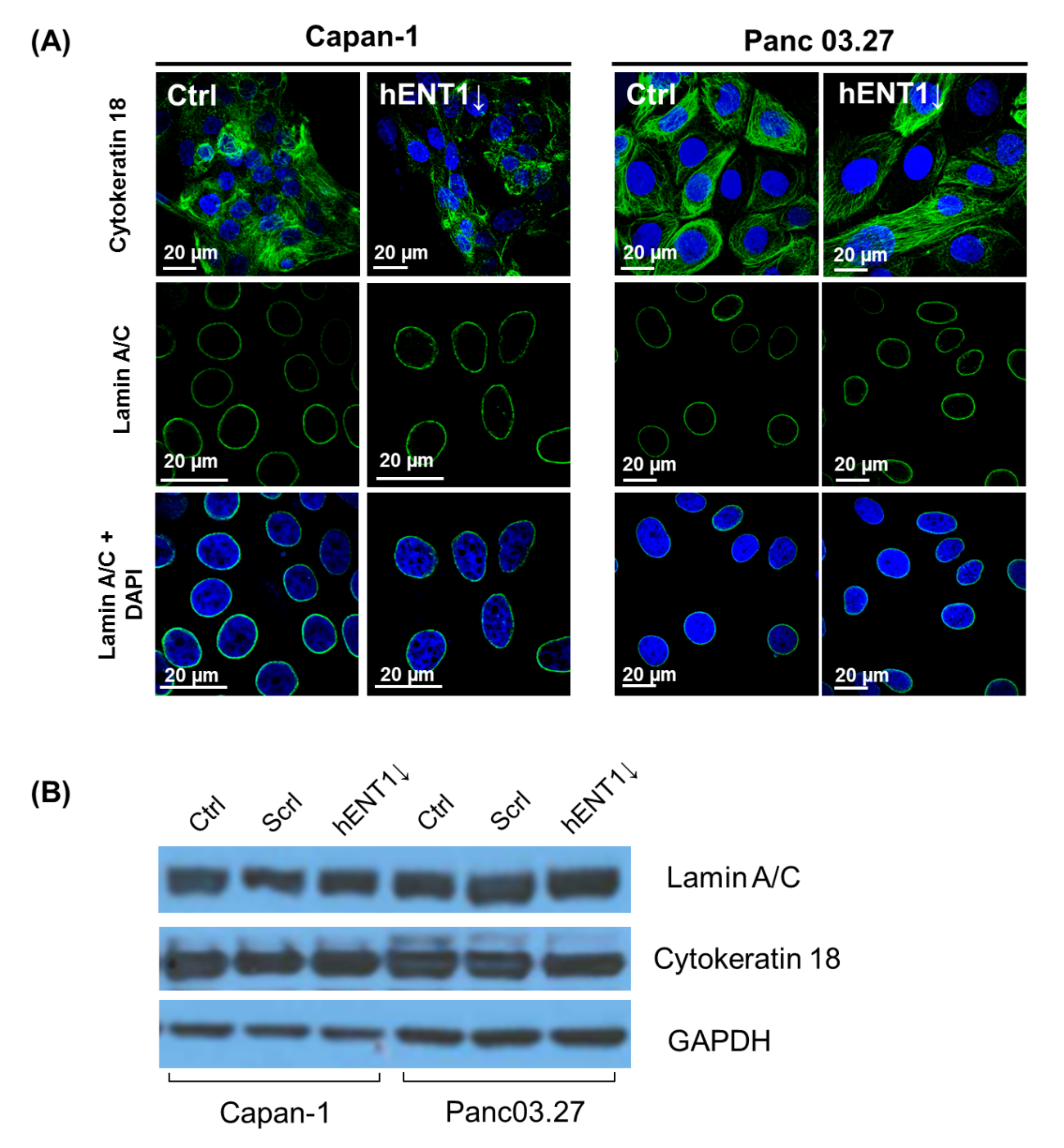


Figure S5. (A) Representative confocal micrographs of pancreatic cancer Capan-1 and Panc 03.27 cells showing cytokeratin 18 (green, top panel), Lamin A/C (green, middle and bottom panels), and nuclei (blue). (B) Western blots of Lamin A/C (74, 63 kDa), cytoketarin 18 (46 kDa) and GAPDH (37 kDa) in control, scramble siRNA transfected, and hENT1 knockdown Capan-1 and Panc 03.27 cells.


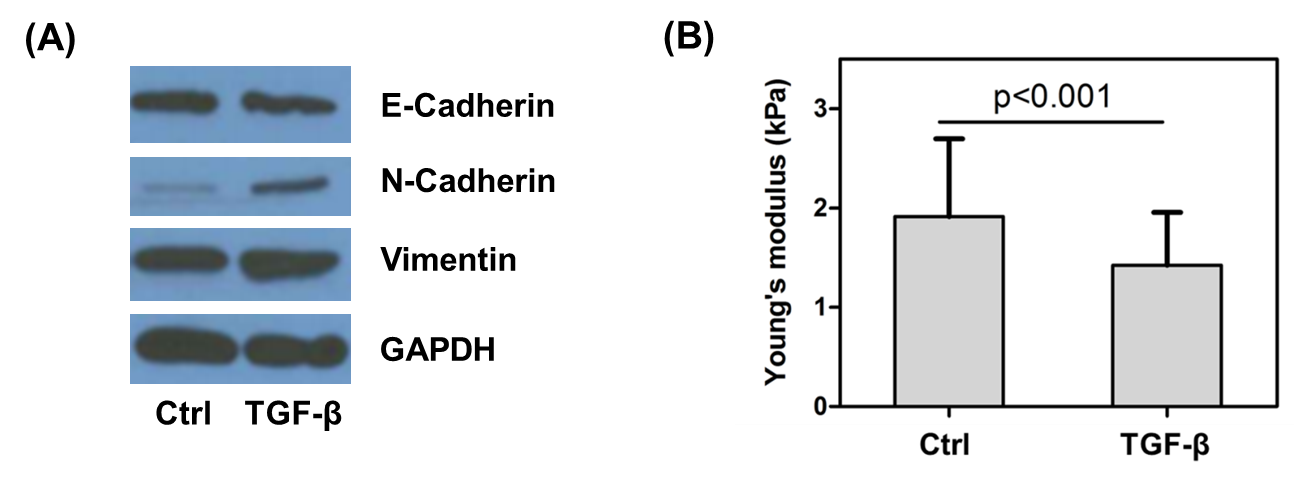


Figure S6 (A) Western blots of E-cadherin (110 kDa), N-cadherin (140 kDa), vimentin (57 kDa), and GAPDH (37 kDa) in untreated and TGF-β treated Panc 03.27 cells, (B) Young’s modulus of untreated and TGF-β treated Panc 03. 27 cells (concentration of TGF-β: 10 ng/ml, exposure for 2 days).

**Force volume imaging and data analysis.**

The Panc 03.27 cells were cultured on 6 cm cell culture dish and all measurements were performed in culture medium at 37˚C. The AFM was equipped with an inverted light microscope (Olympus IX81) so that the cells were constantly monitored. Two different cantilevers were used: one is a pyramid tip cantilever with 10 nm norminal radius in the apex (MSCT-C, Bruker); the other is a 5 μm silica bead-modified cantilever (Novascan Technologies, Inc). The exact spring constant of the cantilever was determined before each experiment using the thermal tune method. Three different 75x75 μm^2^ force-volume maps over 16x16 point grids (256 force-displacement curves per map) were recorded. The applied loading force was set to 100 pN. From force-volume maps, force-displacements curves from points at the cells’ nuclei proximities or cytoplasm were extracted. The Young’s modulus, E, was calculated from obtained force curves based on the Sneddon (data obtained by using MSCT-C cantilever, Eq. S1) or Hertz model (data obtained by using microparticle-modified cantilever, Eq. 1) using Nanoscope analysis program from Bruker corporation.

$$F=\frac{2}{\pi}\frac{E}{\left( 1-\upsilon^{2} \right)}\tan\left( \alpha\right)\delta^{2}$$

where F=force, E=Young’s modulus, ν=Poisson’s ratio (ν=0.5, in this study), α=half-angle of the indenter (i.e. tip radius, α=10nm, in this study), and δ=indentation depth.


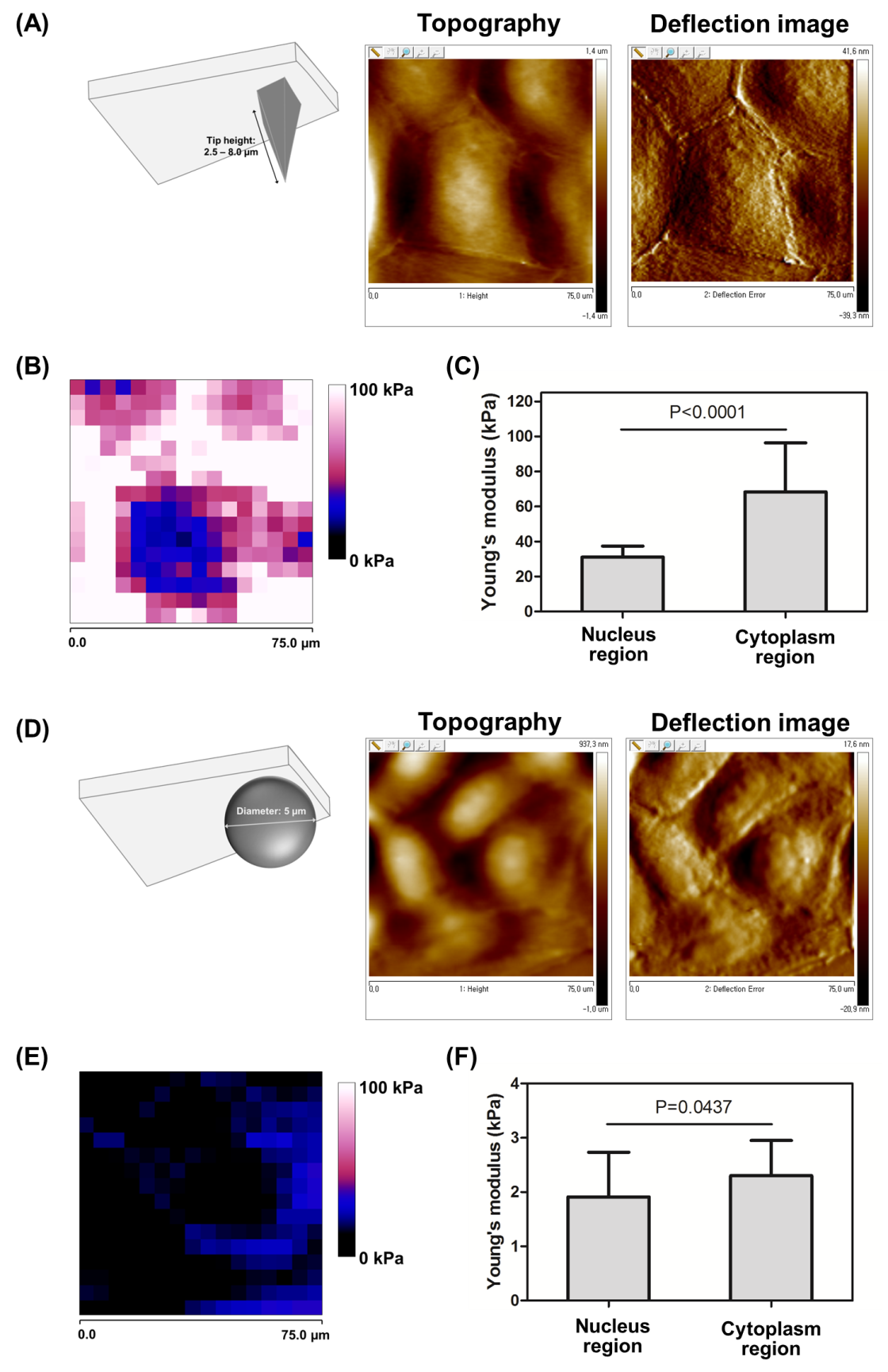


Figure S7. Representative AFM topographic image, deflection image, and corresponding stiffness map of Panc 03.27 cells obtained by using sharp MSCT-C (A, B) and microparticle modified cantilever (D, E). Histograms show (C, F) corresponding stiffness from force volume map (B, E).


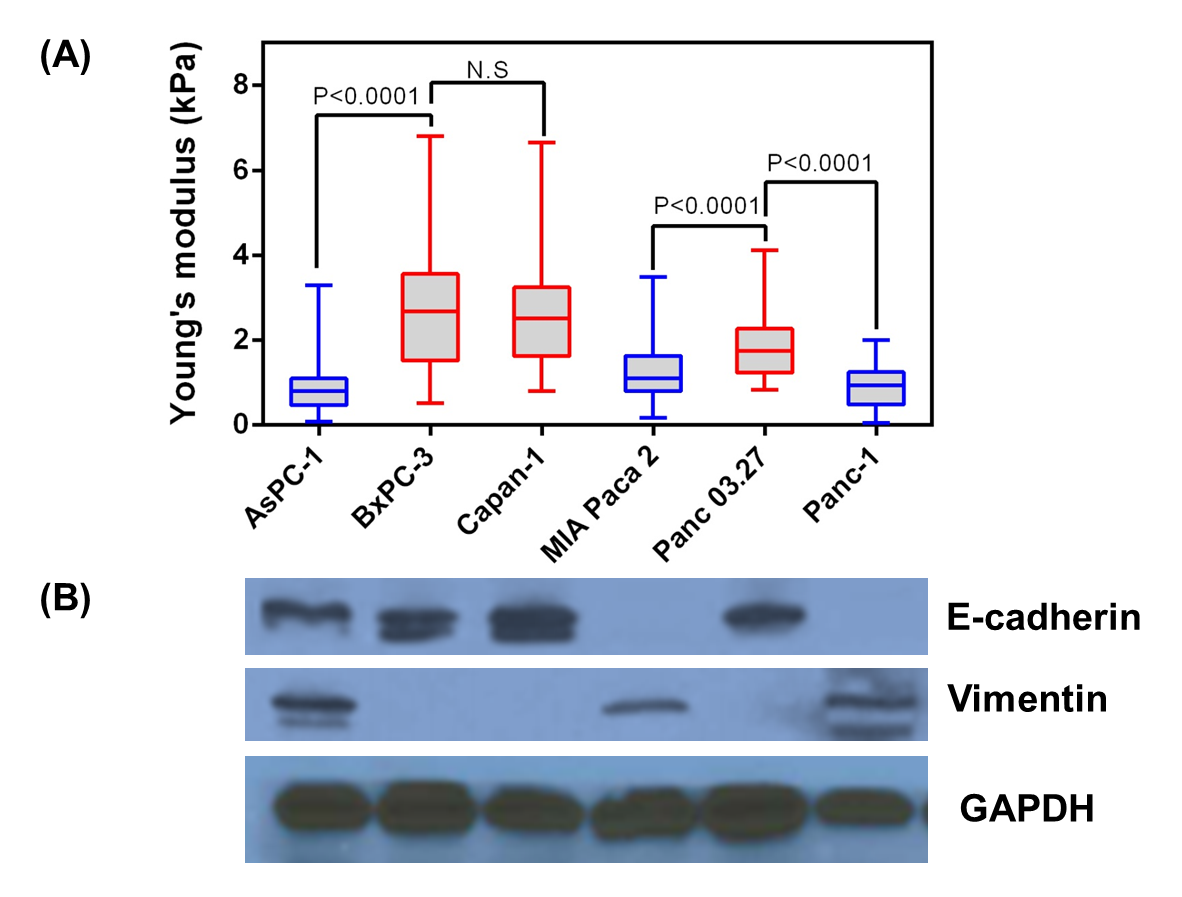


Figure S~~7~~8. (A) Young’s modulus of cells measured by AFM under same indentation force at 100 pN , (B) Western blots of E-cadherin (110 kDa), vimentin (57 kDa), and GAPDH (37 kDa) expressed in six different pancreatic cancer cells.

**References**

1. IUPAC (1997) Compendium of Chemical Terminology. Oxford: Blackwell Scientific Publications.

2. Cross SE, Jin Y-S, Rao J and Gimzewski JK (2009) Applicability of AFM in cancer detection. Nat Nanotechnol 4: 72-73.

3. Hertz H (1882) Ueber die Berührung fester elastischer Körper. crll 1882: 156.

4. McElfresh M, Baesu E, Balhorn R, Belak J, Allen MJ, et al. (2002) Combining constitutive materials modeling with atomic force microscopy to understand the mechanical properties of living cells. Proc Natl Acad Sci USA 99: 6493-6497.
